# Process evaluation of the implementation of the *Unplugged* Program for drug use prevention in Brazilian schools

**DOI:** 10.1186/s13011-015-0047-9

**Published:** 2016-01-07

**Authors:** Pollyanna F. P. Medeiros, Joselaine I. Cruz, Daniela R. Schneider, Adriana Sanudo, Zila M. Sanchez

**Affiliations:** Department of Preventive Medicine, Escola Paulista de Medicina, Universidade Federal de São Paulo, Rua Botucatu, 740, 4° andar, São Paulo, SP Brazil; Department of Psychology, Universidade Federal de Santa Catarina, School of Psychology, Florianopolis, Brazil

**Keywords:** Process evaluation, *Unplugged*, Prevention program, School, Students

## Abstract

**Background:**

Most Brazilian schools do not have a continuous program for drug use prevention. To address this gap, the Ministry of Health adapted the European evidence-based program *Unplugged* to improve the drug use prevention efforts of Brazilian public schools. The aim of this study was to evaluate the process of program implementation in three Brazilian cities among middle school students between 6^th^ and 9^th^ grade (11 to 14 years old).

**Methods:**

Mixed methods were used in this process evaluation study, including focus groups, fidelity forms, and satisfaction questionnaires. Study participants included 36 teachers, 11 school administrators, 6 coaches, 16 stakeholders, and 1267 students from 62 classes in 8 schools.

**Results:**

The 12 *Unplugged* lessons were all implemented in 94 % of the classes. However, only 57 % of the classes were completed as described in the program's manual. The decision to exclude activities because of time constraints was made without a common rationale. Teachers reported difficulties due to the amount of time necessary to plan the lessons and implement the activities. In addition, they mentioned that the lack of support from school administrators was an obstacle to proper program implementation. The majority of students and teachers responded positively to the program, reporting changes in the classroom environment and in personal skills or knowledge.

**Conclusions:**

The *Unplugged* program can be feasibly implemented in Brazilian public schools. However, it is necessary to reduce the number of activities per class and to restructure the format of the standard teaching schedule to ensure that the normal academic content is still taught while *Unplugged* is being implemented.

## Background

Universal prevention in the school environment is a necessary strategy to control substance use among teenagers, particularly because the incidence of initiation of alcohol and other drug use increases significantly between the beginning and the end of adolescence [1]. In Brazil, the average age of initial consumption among students is 13 years old for alcohol and between 13 and 14 years old for other drugs [2], with alcohol and tobacco being the most consumed substances among teens [3]. Based on this data, it is necessary to consolidate interventions to prevent the initiation of substance use and avoid the development of disorders associated with drug use, as highlighted in the three existing United Nations International Drug Control Conventions. In Brazil, there is still a gap in the implementation of drug prevention programs [4], often due to either a lack of teachers who are trained to discuss drug use in the classroom [5] or the lack of support by the school administration [6].

Given the current situation in Brazil, the *Unplugged* Program for drug prevention in the school environment [7] was selected by the Brazilian Ministry of Health to be adapted, implemented, and evaluated in the Brazilian setting. This program is based on a theoretical model of social influence [8] and promotes life skills, provides information on drugs, and develops critical thinking toward social and normative beliefs [9]. The program was selected because of the results obtained in eight countries, which provided evidence of the program’s effectiveness in delaying initiation of tobacco, cannabis, and alcohol use among students between 12 and 14 years of age [10–12].

Despite the program’s proven effectiveness in other contexts, it is still essential to evaluate the implementation process of a program that has been culturally adapted [13]. Process evaluation is an important procedure that assesses the factors affecting program implementation and how the program is received by those involved; the results of process evaluation enable the development of adaptations to enhance the reach and acceptability of the program [14]. In recent years, a large number of studies have noted the importance of evaluating the implementation of preventive interventions in the school environment [15, 16].

Moreover, the use of process evaluations has grown in complexity and importance to now provide a clear definition of what interventions offered to participants based on program fidelity, feasibility, and acceptability [13, 17, 18].

Fidelity determines whether the planned content was implemented effectively and whether the program was accurately administered [19]. Another component analyzed in process evaluation is feasibility, which primarily addresses whether the context and the operational processes involved in program implementation are achievable within the given setting. Finally, acceptability (or satisfaction) is also evaluated, which refers to how the implementation of the intervention met the participants' aspirations and expectations and their satisfaction with the program [20].

This study advances previous studies by assessing all the parties involved in the program implementation and decision-making: government health and education stakeholders, school administrators, program coaches, teachers, and students. Additionally, it uses a mixed methods approach to triangulate data from focus groups and quantitative questionnaires. Finally, this is the first process evaluation study of the *Unplugged* program in a developing country. Evaluating the implementation process of an evidence-based drug school prevention program that was created for use in developed countries will provide invaluable information regarding the implementation of *Unplugged* in a middle-income, developing country. The results can help policy makers understand what elements are essential for the successful adaptation of *Unplugged* in a new context (i.e., from developed to in development countries).

We used three axis of analysis (fidelity, acceptability and viability) as proposed by Rohrbach et al. [16] instead of focusing on only one of the factors. Analyzing multiple implementation variables is necessary because a program with high fidelity but low acceptability by the target population would not be sustainable. We also opted to include the feasibility axis to obtain data on the difficulties faced during the implementation that could or could not be easily resolved, as proposed by Beasley et al. [21] and Goenka et al. [22].

In addition to the process evaluation described in this study, it is important to note that the efficacy of Unplugged in Brazil was also evaluated in a non-randomized controlled trial conducted with 2185 students in 16 public schools in 3 Brazilian cities. Multilevel analyses stratified by age were used to evaluate the changes in the consumption of each drug over time (baseline and 4 months follow up) and between groups (control and experimental). The results indicated that the program seems to have stimulated a decrease in recent marijuana use among students 13 to 15 years old. In addition, students in this age range who received the *Unplugged* program in the classroom maintained drug consumption levels that were similar to those observed before the beginning of the program. On the other hand, students who did not participate in the program showed a tendency to increase their consumption of alcohol, marijuana, and inhalants over the year of the study [23].

The objective of this study was to evaluate the implementation process of *Unplugged* in three Brazilian cities considering its fidelity, feasibility, and acceptability and to analyze how the program activities were implemented, thus providing tools to monitor program quality and generating information needed to adjust the program’s implementation.

## Methods

This study was an evaluation research study that analyzed an implementation process [19]. The research strategy adopted for the analysis was a mixed-methods approach, as proposed by Creswell [24], using quantitative and qualitative instruments to obtain a more holistic understanding [25].

### The intervention

*Unplugged* is an interactive school program based on the Comprehensive Social Influence approach that combines teaching life skills and normative contents in a 50-min weekly lesson that is taught by the school’s current teachers over 12 weeks (Table 1). The design of this intervention has been described by van der Kreeft [7] and is available on the EU-Dap [11] website – www.eudap.net.

**Table 1:** Description of the 12 *Unplugged* lessons by title, activities, and goals, Brazil, 2013

Lesson	Title	Activities	Goals
1	Opening *Unplugged* Program	Presentation, group work, contract management	Introduction to the program, setting of rules for the lessons, reflecting on knowledge on drugs
2	Where do I fit in?	Situation play, game discussion	Clarification of group influences and group expectations
3	Choices – alcohol, risk, and protection	Discussion and work in small groups, collage and drawing	Information on the different factors influencing drug use
4	Does what you think reflect reality?	Presentation, percentage estimates, group work, plenary discussion	Fostering the critical evaluation of information, reflection on differences between own opinion and actual data, correction of norms
5	What we know and what we don’t know about cigarettes	Test, plenary discussion, court	Information on the effects of smoking, differentiation between expected and real effects and short-term vs. long-term effects
6	Express yourself	Game, plenary discussion, group work	Adequate communication of emotions, distinguishing between verbal and nonverbal communication
7	Position yourself in the world and in your life	Plenary discussion, group work, role play	Fostering assertiveness and respect for others
8	New in the area!	Role play, game, plenary discussion	Recognition and appreciation of positive qualities, acceptance of positive feedback, practicing and reflecting on getting into contact with others
9	Drugs – Get informed	Group work, quiz	Information on the positive and negative effects of drug use
10	Coping strategies	Presentation, plenary discussion, group work	Expression of negative feelings, coping with challenges
11	Problem solving and decision making	Presentation, plenary discussion, group work, homework	Structured problem solving, fostering creative thinking and self-control
12	Goal setting	Game, group work, plenary discussion	Distinguishing between long-term and short-term objectives, feedback on the program and the process during the program

Source: Adapted from the Teacher's Handbook (www.eudap.net)

The program includes the following supporting resources: the teacher’s handbook, which provides information on each classes' procedures, objectives, necessary materials, tips, and planned activities and the student's workbook, which includes the activities that will be conducted by the teacher in each class.

In Brazil, the English versions of these materials were translated into Portuguese, including the adaptation of idioms and the modification of information to be more appropriate to the Brazilian context. Teachers attended a 16-h training session that was facilitated by coaches from the Ministry of Health who had been trained by the European developers of the program [7].

At the end of each lesson, the teachers completed a fidelity form (monitored class by class) to assess the dose of the program offered (quantity of program activities performed), following the structure of the EU-Dap monitoring [26]. To ensure fidelity, teachers were supervised every three weeks by the Ministry of Health coaches through in-person meetings. During these meetings, the coaches confirmed that the fidelity forms were being completed, helped teachers plan the lessons, checked the delivery of lessons, and solved potential challenges that the teachers might be facing, thus standardizing the implementation throughout all the participating schools.

### Participants in the program

In Brazil, the implementation of the *Unplugged* Program included 5 stages: establishment of the state- and municipal-level policies with health and educational stakeholders to enable implementation in public schools; selection of the schools; training of the coaches by the program developers; training of the teachers by the coaches; and monitoring and evaluation of the program implementation in schools.

During the second school semester of 2013, eight Brazilian schools used the *Unplugged* Program in the classrooms of 6^th^ to 9^th^ grade students. The program was implemented in 3 Brazilian cities: São Paulo, São Bernardo do Campo (both in São Paulo state, southeastern region of Brazil) and Florianópolis (Santa Catarina state, southern region of Brazil). Overall, the program reached 1833 students in 62 classes and was taught by 36 teachers. The distribution by region was 1210 students, 40 classes, and 15 teachers in São Paulo state and 623 students, 22 classes, and 21 teachers in Santa Catarina state.

The participating schools were selected and nominated by the State and Municipal Departments of Education in the regions involved based on the lack of an active program for drug use prevention for 6^th^ to 9^th^ grade students and the absence of explicit problems with drug trafficking. Each school principal recommended the teachers who would participate in the program, and each teacher could apply the program in up to 3 of their classes. The program was applied weekly in each of the selected classes to all the students present in the classroom.

It is important to note that *Unplugged* is a universal prevention program that is indicated for groups in which the prevalence of initial drug experimentation is low [27]. The schools were selected due to their "normal profile," meaning they did not have a noticeable presence of drug consumption by the students nor explicit drug traffic. Furthermore, the schools did not have an ongoing formal drug use prevention program to avoid any contamination of the results. As the program had not been evaluated in groups with a high prevalence of drug use, it did not make sense to choose schools with a high drug profile for the first implementation and evaluation in Brazil. This decision to select normal profile schools was made by the health and education governmental secretariats as the Brazilian schools that were vulnerable to drug trafficking would have presented more challenges to the adaptation of the program to Brazilian culture.

### Study participants

The results of this study describe the process evaluation of the classroom intervention. The sample of participants for this study was selected from the group of participants in the program.

Five groups of people who were involved in different stages of the program implementation process participated in the study: students, teachers, stakeholders (members of the state and municipal departments of health and education), school administrators (principals and pedagogical coordinators), and coaches (Table 2).

**Table 2:** Data collection techniques, number of respondents, type of participants, instruments, axis and variables used in the process evaluation of the *Unplugged* Program implementation, Brazil, 2013

Type	Technique	Participants	Timing	Instrument	Axis of analysis	Variables
Qualitative	Focus Group (13 focus groups, *n* = 100 participants)	Teachers (*n* = 13), Stakeholders (*n* = 16), School Administrators (*n* = 11), Coaches (*n* = 6), Students (*n* = 54)	At the end of the 12 *Unplugged* lessons	Semi-structured script/guide	Acceptability, Feasibility; Fidelity	Development of prevention activities in the school environment; aspects that make the implementation process difficult or easy; sustainability of the program; opinions about the lessons, opinion about the perceived results; evidence of classroom adaptations.
Quantitative	Fidelity Form (*n* = 655)	Teachers (*n* = 36)	At the end of each lesson taught, weekly	Self-report questionnaire with open-ended and closed questions	Fidelity; Feasibility	Total number of students participating in each class; Activities conducted; Time spent in each activity; Class engagement; Elements to modify the interaction between students and teachers.
	Satisfaction Questionnaire (*n* = 1294)	Students (*n* = 1267) and Teachers (*n* = 27)	At the end of the 12 *Unplugged* lessons	Self-report questionnaire with open-ended and closed questions	Acceptability	Positive aspects of the program; Negative aspects; Interest in maintaining the program; Improvement in the relationship between teachers and students; Improvement in the relationship among students.

The participants in the focus groups were 54 students (6 groups), 13 teachers (2 groups), 16 stakeholders (2 groups), 11 school administrators (2 groups), and 6 coaches (1 group) divided by category and by Brazilian State. The participants were randomly selected from the entire population of participants and consisted of those who were available on the day and time scheduled by the research team. Ten participants were invited to each focus group, and an average of seven agreed to participate in each group. The refusals to participate were mainly due to scheduling conflicts on the days when the focus groups were scheduled.

Students and teachers completed the satisfaction questionnaires anonymously one week after the end of the program. Only the students and teachers who were present in the school that day responded to the instruments; therefore, the questionnaires were not completed by all the participants in the program (participation rate: 75 % for teachers and 71 % for students).

### Techniques for data collection and instruments

Qualitative and quantitative (questionnaires) methods were used to collect the data for this study. Further details on the techniques, instruments, variables, axis of analysis and the participants involved are presented in Table 2. The instruments used in the quantitative analysis were the Portuguese versions of the same instruments used by the EU-Dap team in their process evaluation of the *Unplugged* program in Europe [28]; these instruments are available at www.eudap.net. The Brazilian research team created the qualitative guides for the focus group interviews.

We opted to use a fidelity model based on dosage that was proposed by the *Unplugged* developers. Fidelity was defined by the number of lessons taught, the time spent per lesson, the number of activities taught per lesson and the number of the potential participants present in each class as outlined in the EU-Dap process monitoring manual and dissemination guide [26, 27].

### Data analysis

#### Quantitative data analysis

Three sets of data were analyzed using descriptive statistics: the student satisfaction questionnaire, teacher satisfaction questionnaire and the fidelity form (monitoring of each lesson). Each of the questionnaires used in the process evaluation was submitted to a specific database through an online system. Qualitative variables were described by number, percentage and 95 % confidence interval, whereas the quantitative variables were described by the mean and minimum and maximum values. The distribution of the students’ and teachers’ perceptions of the *Unplugged* program’s immediate results were evaluated by Chi-square test.

#### Qualitative data analysis

The recordings of the focus groups were fully transcribed and underwent content analysis using the Grounded Theory approach [29] as the theoretical reference. In this approach, categories are developed from the results of the data, avoiding “a priori” theories to generate explanations that are based in reality. Study data were then categorized conceptually using codes, a process that organizes data into an applicable understanding of the investigated phenomenon [30]. Data coding creates data segments that are defined by the most concise and objective categories to summarize and represent the meaningful components of the transcribed text [31].

After the content analysis, the focus group materials were classified into five broad themes; this article focuses on the results of the difficult and easy aspects of the program and the immediate results of the implementation process. The qualitative analysis was conducted with the support of the computer program NVivo version 10 [32]. Quotes from the focus groups are presented in the results section with a reference to the group from which they were extracted.

The data were analyzed separately, and then the results of the focus groups, the fidelity forms, and the satisfaction questionnaires were triangulated to address the fidelity, acceptability, and feasibility of the program’s implementation.

### Ethics

This study was approved by the Ethics in Research Committees at the University of São Paulo (#473.498) and the Federal University of Santa Catarina (#711.377); all the project stages were compliant with the Declaration of Helsinki. Informed consent to participate in the study was obtained from all the participants.

## Results

### Fidelity

Of the 744 lessons planned (12 lessons in 62 classes), 698 lessons were actually taught and 655 had the corresponding fidelity form completed by the teachers after the end of the lesson. Based on this data, we generated a fidelity index (dose) of 94 % (CI 95 % 92%–95 %) for the number of lessons implemented (698/744) and 94 % (CI 95 % 92 %–95 %) for the monitoring of the implementation (655/698).

Although the vast majority of the lessons were taught in the 62 classes, only a small percentage of the lessons included all the activities proposed in the teacher's handbook. Lesson 11 was completed by only 21.7 % of the classes. Moreover, Table 3 shows that the lessons reached an average of 80 % of the expected students.

**Table 3:** Results on the fidelity and reach of the *Unplugged* program based on the 655 completed fidelity forms, Brazil, 2013

	Fidelity (dose)					Reach		
	Applied lessons					Students present at the lesson		
	N	%	Full lesson^(a)^ (43)	Mean (minutes)	Min-Max (minutes)	Mean	Min-Max	%^(b)^
Lesson 1	62	100.0	59.7 [46.4–71.9]	68	40–120	27	13–40	91.7
Lesson 2	62	100.0	54.0 [40.1–66.0]	63	40–95	27	15–40	91.5
Lesson 3	61	98.4	64.4 [50.6–75.8]	69	40–95	27	12–40	92.7
Lesson 4	61	98.4	48.2 [34.6–60.7]	64	40–105	26	14–40	85.8
Lesson 5	61	98.4	39.7 [27.1–52.7]	69	40–115	27	17–40	80.1
Lesson 6	62	100.0	24.6 [14.2–36.7]	62	40–95	26	18–40	84.9
Lesson 7	58	93.5	42.9 [30.2–56.8]	60	30–95	26	14–40	83.1
Lesson 8	55	88.7	61.5 [47.7–74.6]	64	40–110	25	15–40	75.8
Lesson 9	59	95.2	45.1 [32.7–59.2]	60	20–105	25	15–40	72.4
Lesson 10	54	87.1	42.3 [29.2–56.8]	61	40–105	26	17–40	74.4
Lesson 11	50	80.6	21.7 [11.5–36.0]	62	40–105	25	16–40	62.9
Lesson 12	53	85.5	52.9 [38.6–66.7]	61	20–105	25	12–40	69.9
*Mean Unplugged*	58	93.8	46.4 [33.3–60.1]	64	20–120	26	12–40	80.4

(a) % of classrooms in which all the planned activities in the lesson were delivered to the students

(b) % = number of students present at the lesson / number of students expected to be present * 100

Another important aspect of fidelity is the comparison between the actual time spent by the teacher applying each *Unplugged* lesson and the expected lesson time. The orientation provided during the teacher training stated that all the program lessons should be performed within the time allotted for one lesson, which in São Paulo corresponds to 50 min and in Santa Catarina to 45 min. According to the data from the fidelity forms (*n* = 655), 56.9 % of the lessons were performed in the time allocated for one lesson; 11.5 % of the lessons lasted for one and a half lessons; 23.9 % of the *Unplugged* lessons lasted for the duration of two lessons; and 7.7 % of them lasted more than two full lessons.

Furthermore, data from the focus groups reinforced the notion that it was not feasible to implement a complete *Unplugged* lesson as proposed in the handbook in the time allocated for one lesson. This conclusion was unanimous among the teachers.

*“This issue of having only 45 minutes, I've already told the coach several times; it is not feasible.”* (Teacher)

Many of the activities in the program were not used or were only briefly performed with the students, indicating that the objectives of the lessons and the program may not have been reached.

*“I had to cut things out before starting the class. [I’d ask myself,] “What am I going to leave out?” This 10-minute opening will last 2 minutes. The energizer* [group dynamics to stimulate interaction among students in the classroom] *is out. This here, out… That's what I did!”* (Teacher)

School administrators and teachers thought that the increased time necessary to cover the *Unplugged* lessons was perhaps due to the Brazilian culture and the learning conditions of several students, which affected the program activities. This finding indicates a need for the program to be better adapted to the Brazilian school setting*.*

*“T1- [50 minutes] is how long students in Europe take to complete the activity. Here, maybe because of our culture, until we arrive, until we organize the classroom, until students are seated…*

*T2- (…) Here, a 50-minute lesson ends up taking an hour and a half, an hour and forty minutes.”* (School administrators)

*“Then, there is also our students' difficulties in writing. Our students cannot write. In the 6*^*th*^*grade there are students who are almost illiterate. There are questions that they need to answer, and if you let them do it at their own pace, it would take a long time for them to answer.” (*Stakeholder)

### Feasibility

The main difficulties and successes of the program according to the teachers, school administrators, and coaches were identified through the focus groups.

Most of the difficulties experienced in the program implementation process were described by the teachers, who identified 3 main obstacles that affected the feasibility of the implementation: time spent planning lessons, lack of material resources, and the undermining of the students’ standard curriculum content.

The lack of time dedicated to preparing the program activities and to appropriately implement them was strongly highlighted by the teachers because they added a classroom activity to their previous ones, and there was no reduction in their other responsibilities.

*“[Unplugged] is not something that you can start applying right away. The teacher needs to study these lessons, to prepare them. For certain lessons, they need materials, even some of the energizers need it, so they need preparation. In this sense, I notice that [the teachers] are a bit overloaded because in addition to Unplugged they need to cover the curriculum, and their time is limited.”* (School Administrator)

The analysis of the coaches’ responses clearly showed that the teachers were overwhelmed with the excessive amount of program protocols required. The teachers themselves also mentioned this issue:

“*This moment of having a meeting with everyone is impossible because at this time of the year we don't have the time to do that. Sometimes someone in the school asks 'can you talk to me now?', [and I say] 'no, I can't'! It’s impossible.”* (Teacher)

There was a discrepancy between the teachers and the school administrators regarding the support provided by the school. School administrators highlighted more than once that they offered full support in terms of pedagogical materials for the development of the program, whereas the teachers mentioned that a lack of pedagogical materials and resources to make copies of the material was a barrier to the feasibility of the program. According to the teachers, not all the school administrators offered support for the implementation of the program.

The content of the normal academic curriculum delivered was also affected in the students whose teachers taught *Unplugged* because the teachers had to replace the curriculum lessons with the *Unplugged* lessons. Teachers unanimously felt that their academic curriculum was not fully covered, with parts of it being only superficially taught to the students during the semester; this may have led to gaps in the students’ education. Teachers feared being blamed by the students for these gaps.

*“Another important aspect is that they [teachers] had to stop teaching the content of their subjects, and that is it. If something is added, something else has to be removed, and that is what happened.”* (Coach)

However, the stakeholders did not mention the pressing need to adapt the logistics of the program for it to be sustained in the schools, demonstrating a clear discrepancy between the practical perceptions of the implementation by the stakeholders, school administrators, and teachers. Nevertheless, the stakeholders did highlight the need to incorporate *Unplugged* into the regular classroom curriculum.

*“Usually the school project always includes prevention actions (…) in this sense, I think that somehow (…) we can think of structuring [Unplugged] to be a part of the regular classroom curriculum.”* (Stakeholder)

Teachers highlighted the need to make basic adaptations to the program for it to become part of the school curriculum. According to them, the training they received, the support by the school administration, and the presence of coaches were the main factors that facilitated the implementation of *Unplugged*.

### Acceptability

The results obtained in this study showed a satisfactory acceptability of the program based on the triangulation of data from the focus groups, fidelity forms, and satisfaction questionnaires, although there were some discrepancies between the data from the students and from the teachers. For 69.4 % of the students, the program helped them answer personal questions. Of the teachers, 88.9 % said that it improved their knowledge and skills on drugs and prevention. The program satisfaction in the school setting was clearly observed, as shown in Table 4. The *p* value indicates that for all the questions, both teachers and students tended to choose the positive answer option.

**Table 4:** Students’ and teachers’ perceptions of the immediate results of *Unplugged* based on data from the satisfaction questionnaire, Brazil, 2013

		Opinion						
		Positive		Neutral		Negative		
	Perceived results	N	%	N	%	N	%	*p* ^(a)^
Students (*N* = 1267)	Helped answer personal questions	867	69.5	302	24.2	79	6.3	<0.001
	Changed the way in which the student sees her/himself	647	52.0	341	27.4	257	20.6	<0.001
	Improved knowledge of drugs	1085	87.6	107	8.7	46	3.7	<0.001
	Improved relationship with colleagues	510	40.8	506	40.5	234	18.7	<0.001
	Improved relationship with teachers	519	41.8	477	38.5	244	19.7	<0.001
Teachers (*N* = 27)	Improved knowledge and skills regarding drugs and prevention	24	88.9	1	3.7	2	7.4	<0.001
	Enriched teaching skills	24	88.9	1	3.7	2	7.4	<0.001
	Improved relationship with students	25	92.6	1	3.7	1	3.7	<0.001
	Improved relationship among students	21	77.8	6	22.2	0	0.0	<0.001

(a) Chi-square: comparison of the % of Positive, Neutral, and Negative responses

One of the study’s notable findings was the improvement in the relationship both between teachers and students and between students in the classroom. This result was identified from qualitative and quantitative data, namely teachers’ and students’ focus groups discussions and satisfaction forms (Table 4).

According to the teachers' opinions in the fidelity forms, the level of students' interest in the 12 *Unplugged* lessons in the 62 classes was, on average, very high or high in 48.2 % of the lessons (*n* = 316), moderate in 46.5 % (*n* = 304), and absent/very low in 5.2 % (*n* = 34).

An indirect assessment of the acceptability of the program by the teachers was obtained from their reported level of comfort in teaching the lessons. Teachers provided this information at the end of each lesson on the fidelity form. Based on the data from these forms, most of the teachers described, on average, a high to very high comfort level (51 %, *n* = 334). In 45 % (*n* = 295) of the cases, there was a moderate level of comfort, and no comfort was reported in 4 % of the cases (*n* = 26).

Finally, the teachers and students were asked the direct question, “how satisfied have you felt with participating in the program?” The responses to that question demonstrated a positive picture for maintaining the program in schools. Almost all the teachers (92.6 %) said that they were “satisfied or very satisfied” with the program. Students had a similar positive perspective, although a smaller proportion of them were “satisfied or very satisfied” (77.9 %).

When asked which lessons they liked the most, the students said that their interest in the lessons depended on the topic discussed. Although their interest was usually high, their responses showed that the lessons with information on drugs and making choices in groups were the ones that generated the most interest.

*“I liked the one [lesson] in which we either followed a group's decision or not because you don't have to do what the group does to join it, to be like them.”* (6^th^ and 7^th^ grade students)

*“I liked the exercise where there were two groups and there were also judges. One was about defending alcohol and tobacco and what alcohol and tobacco did.”* (6^th^ and 7^th^ grade students)

Because the students enjoyed the *Unplugged* activities, they suggested that there should be a specific class for the program with more time allocated to it than what was planned in the pilot phase. They also supported the continuation of the program in the following year.

*“I think that there should be more time for the lessons; there was a lesson we started and then the bell rang, and we had to finish it in the following class. We should have a specific class for Unplugged.”* (8^th^ and 9^th^ grade students)

Finally, 76.5 % (CI95% 74.0; 78.8 %) of the students reported that they would like to have a program similar to *Unplugged* in the following school years.

## Discussion

This study presents the process evaluation of a European evidence-based drug use prevention program that was created in developed countries and implemented in Brazil, a Latin American, middle-income, developing country. The process evaluation results of this study, in addition to the outcome results, will inform stakeholders’ decisions regarding the program’s maintenance and expansion in Brazilian public schools by providing data on the program’s feasibility and acceptability as well as possible adaptations to increase its fidelity.

Researching the acceptability of the program in the different groups involved in *Unplugged* was essential for its appropriate implementation in schools, as a better understanding of the acceptability is an important tool in the development of management strategies to expand drug prevention interventions. User satisfaction is a component of social acceptability and is necessary for a program to be successful [18]. Acceptability represents the approval of a service by its target population, and the acceptability of *Unplugged* was evident in the findings of this process evaluation.

The assessment of the feasibility of the program indicated that its implementation was possible despite the difficulties highlighted by the teachers. The teachers emphasized that the program required time to plan the activities and to prepare the classroom, which was not incorporated into the schools' regular curriculum. This affected the teachers' regular curricular activities and overburdened them with new activities. The data showed that the time per lesson and, consequently, the total time needed to apply the program in a school year were elements that had to be factored into the regular classroom schedule as only 57 % of the lessons were effectively taught within the expected time. We found that the teachers randomly excluded activities when they realized that they would not able to teach the complete lesson, which suggests that it would be useful for the program developers to identify the core elements of a lesson that should be taught by the teacher even when time is constrained.

The fidelity evaluation conducted in this study, as proposed by the developers of *Unplugged* [26, 27], focused on the dose (quantity of lessons and of activities per lesson) and identified the schools’ ability to implement all the 12 program lessons in a school semester. However, it was clear that one Brazilian class hour (45 to 50 min) was not enough for all the planned activities (approximately 3 to 5 per lesson) to be completed in some of the classes. The decision regarding which activity to exclude when time was limited was made individually by each teacher, with no common rationale among the teachers; this difference in the implemented activities could have affected the core characteristics of the program, as previously described by Kreeft et al. [28]. However, it is important to highlight that in the first randomized controlled trial of *Unplugged* in European countries, almost half of the classes were not delivered to the experimental group, and despite this fact, the program revealed effectiveness in reducing alcohol and tobacco consumption among students [11]. Although several authors have argued that implementation fidelity guarantees the non-mischaracterization of a program and also its effectiveness [33], in our case, we can assume that the program fidelity ensured dosage control. Furthermore, we found that this study showed greater fidelity than the European studies that demonstrated the program’s success.

The only way to guarantee the sustainability of the program would be to incorporate it into the school curriculum; this would ensure that the program activities would not interrupt the time allocated for teachers' regular subjects. However, it is important to note that the lack of time reported by the teachers to implement the normal school program is not unique to this setting. A study evaluating the process of implementation of a depression prevention program for teenagers in classrooms showed similar conflicting demands of teachers' time between the program and the regular curriculum. In addition, it revealed a culture in which exclusively academic activities were more important than the personal, social, and health-related activities of the students, which negatively affected the implementation of the program in schools in England [34].

According to Sloboda et al. [35], when there is a lack of a structural and curricular program, the intuitive development of drug prevention activities in the classroom or debates with former drug users are often what is observed. These types of interventions contradict the current scientific evidence, which shows the ineffectiveness of these models when used in isolation and out of context. *Unplugged* was adopted to fill this organizational gap as it also aligned with the guidelines described in the “Integrated Policy of Attention toward Alcohol and Drug Users" written by the Brazilian Ministry of Health [36]. These guidelines define prevention as a process of planning and implementing multiple strategies geared towards reducing specific vulnerability and risk factors and strengthening protective factors. However, initiatives integrating health and education are still being developed. According to Deschesnes et al. [37], for a prevention program to become part of the school structure, it is necessary to include it in the school’s yearly planning; this requires a systematic coordination by school administrators as well as integrated and intersectional actions and a political and financial commitment by the decision makers. Once these stages are established, the evaluation process begins with the goal of identifying potential adaptations to the initial program that could facilitate the improvement of the intervention.

*Unplugged* has proved to be an intervention that is well accepted among the participants directly involved (students and teachers) in addition to being effective in European countries. Furthermore, the program is structured using interactive techniques, which allows for the development of life skills. In a meta-analysis that evaluated school prevention programs, Tobler et al. [38] found that interactive prevention models were more effective than non-interactive ones. An interactive approach provides contact and opportunities for the exchange of ideas among participants, strengthening the skills needed to refuse drugs through group dynamics. Moreover, according to a study conducted by Sanchez et al. [39], information about drugs is also a necessary form of prevention. However, if the school provides information that is not reinforced by the parents, the knowledge gained can lose its preventive effects; information in isolation that is disconnected from activities that reinforce protective factors and reduce risk factors is not effective in preventing drug consumption [40].

*Unplugged* provided a distinct form of intervention in the school environment. It also influenced the perceived relationship between the teachers and the students as well as between students in the same classroom, independent of the results on drug use prevalence (which were not the focus of this article). These results suggest that it would be important to include classroom environment outcomes as secondary outcomes in the next randomized controlled trial of *Unplugged*.

This is the first study of the *Unplugged* program that focuses on the implementation process and not on the program’s effectiveness results. This research expands the program’s literature on transcultural adaptation as *Unplugged* was designed for developed European communities and not for developing countries. Considering the unique nature of the public schools in one of the most unequal countries in the world, the absence of acceptance among stakeholders, school directors, teachers and students would have resulted in the quick termination of the program in schools despite the federal government’s support for its implementation.

Although the study presents relevant data, there are inherent limitations of the methods that should be considered. One of these limitations was that the participating schools were not randomized; they were nominated by the department of education in each of the participating cities. In addition, the school staff selected the students who participated in the focus groups, and this could have prevented the representation of the general student body in the study. Finally, the high burden of demands on the teachers may have reduced their willingness to complete the satisfaction questionnaire that was collected in the last school week of 2013.

There is no way to conclude whether the schools included in this study differed from those not included. What we can confirm is that the schools included had a similar drug use pattern to the ones described in the last national survey on drug use by middle and high school students in Brazilian capitals [2, 23], which seems to suggest that the participating schools exhibited the national average consumption for the selected age. Data on drug use in our sample were not presented in this article, but that data have been submitted for publication in another article, as previously mentioned.

## Conclusion

The relevance of this study is that it deepened the available scientific knowledge on drug use prevention programs in the school environment; in particular, it generated results that can inform decision-making regarding the implementation of *Unplugged* in schools in developing countries.

The dose of *Unplugged* that was offered in the classrooms was satisfactory as almost all the classrooms received 12 lessons. However, the number of activities completed in each class was inadequate; almost half of the lessons were not completed during the 45- to 50-min class period. All the studied groups described positive perceived results on school environment, and teachers and students mentioned an improvement in their relationships. However, a significant challenge reported by the study participants was the inability of the teacher to provide the same quality teaching of their normal curricula; teachers had to deliver the *Unplugged* lessons during their regular class time and did not have additional hours to teach the subjects that were replaced by the *Unplugged* activities.

Based on the analyzed results, we suggest three adaptations that could improve the implementation of the program. The first recommendation is to adapt the number of activities per class with the support of the developers to allow each class to be fully executed and to prevent teachers from having to randomly exclude activities. Second, we recommend restructuring the general workload of the teachers who implement the *Unplugged* program to avoid using the regularly scheduled class time for the *Unplugged* activities. Finally, a randomized controlled trial that includes school environment outcomes in the analysis, such as the relationship between teachers and students, is recommended.
